# Structure/Function Analysis of Truncated Amino-Terminal ACE2 Peptide Analogs That Bind to SARS-CoV-2 Spike Glycoprotein

**DOI:** 10.3390/molecules27072070

**Published:** 2022-03-23

**Authors:** Robert T. Mackin, J. Vincent Edwards, E. Berk Atuk, Noah Beltrami, Brian D. Condon, Janarthanan Jayawickramarajah, Alfred D. French

**Affiliations:** 1 United States Department of Agriculture, Agricultural Research Service, Southern Regional Research Center (USDA-ARS-SRRC), New Orleans, LA 70124, USA; rmackin87@gmail.com (R.T.M.); brian.condon@usda.gov (B.D.C.); al.french@usda.gov (A.D.F.); 2 Department of Chemistry, Tulane University, New Orleans, LA 70118, USA; eatuk@tulane.edu (E.B.A.); nbeltram@tulane.edu (N.B.); jananj@tulane.edu (J.J.)

**Keywords:** SARS-CoV-2, COVID-19, cyclic peptide, helical peptide, surface plasmon resonance

## Abstract

The global burden of the SARS-CoV-2 pandemic is thought to result from a high viral transmission rate. Here, we consider mechanisms that influence host cell–virus binding between the SARS-CoV-2 spike glycoprotein (SPG) and the human angiotensin-converting enzyme 2 (ACE2) with a series of peptides designed to mimic key ACE2 hot spots through adopting a helical conformation analogous to the N-terminal α1 helix of ACE2, the region experimentally shown to bind to the SARS-CoV-2 receptor-binding domain (RBD). The approach examines putative structure/function relations by assessing SPG binding affinity with surface plasmon resonance (SPR). A cyclic peptide (c[KFNHEAEDLFEKLM]) was characterized in an α-helical conformation with micromolar affinity (KD = 500 µM) to the SPG. Thus, stabilizing the helical structure of the 14-mer through cyclization improves binding to SPG by an order of magnitude. In addition, end-group peptide analog modifications and residue substitutions mediate SPG binding, with net charge playing an apparent role. Therefore, we surveyed reported viral variants, and a correlation of increased positive charge with increased virulence lends support to the hypothesis that charge is relevant to enhanced viral fusion. Overall, the structure/function relationship informs the importance of conformation and charge for virus-binding analog design.

## 1. Introduction

The recent coronavirus (SARS-CoV-2), which was identified in Wuhan, China, at the end of 2019 [1,2], has devastated the world in terms of lives lost and economic impact. As of January 2022, more than 312 million people have been infected globally and over 5.5 million have died as a result of COVID-19 [3], the disease caused by the novel coronavirus. Early research found that the virus shares nearly 80% similarity in terms of its sequence with the coronavirus that caused the pandemic in 2003 (SARS-CoV) [4,5], yet recent investigations using SARS-CoV viral inhibitors have shown that the differences between the two are great enough to prevent these treatments from being effective at neutralizing SARS-CoV-2 [6].

The mechanism for viral entry into host cells is now well-established and follows a similar route as SARS-CoV, where the SARS-CoV-2 spike glycoprotein (SPG) receptor-binding domain (RBD) fuses to the host receptor angiotensin-converting enzyme II (ACE2), found in epithelial cells in the lungs, nostrils, intestines, and other places in the human body [7,8]. Recent studies have shown that key differences in the viral protein sequences result in a higher binding energy between ACE2 and SARS-CoV-2, which has been shown to make the novel coronavirus more virulent than its 2002–2003 predecessor [9,10]. Once bound, the SPG is primed by host proteases that cleave the SPG, after which the internalization of the virus into the host cell occurs [11].

The recent development and implementation of multiple vaccines has significantly decreased the infection rate and transmission of the SARS-CoV-2 virus worldwide [12]. Unfortunately, variants have emerged in the form of spike protein RBD mutations that have been shown to be more virulent and potentially deadlier than the wild-type (WT) SARS-CoV-2 [13,14,15]. These variants have appeared in densely populated regions of the world, where the virus is able to replicate and transmit to new hosts. Initially, the mutated strains of highest concern contained the N501Y mutation on the RBD, which has appeared in three locales, including the UK (B.1.1.7 or N501Y.V1), South Africa (B.1.351 or N501Y.V2), and Brazil (P.1 or N501Y.V3). Each has shown improved ACE2 fusion [16,17,18]. Additionally, the “Delta” variant (B.1.617.2), originating in New Delhi and containing the P681R substitution beside the S1/S2 furin cleavage site, has been shown to increase cleavage efficiency, and more recently, the emergence of the “Omicron” variant (B.1.1.529), originating in Botswana, contains more mutations than any variant of concern previously seen [19,20], with further evidence suggesting that the longer the pandemic persists, the more mutations will be introduced into the sequence. Overall, these mutations have resulted in an increased transmission rate and a higher occurrence of COVID-19 in the affected regions [21,22,23], and the longer the virus persists, the greater the potential for the emergence of deadlier mutations. Moreover, the appearance of these mutated strains poses an even greater risk to the global community in that recent studies have shown that the variants have some or complete resistance to antibodies used for COVID-19 treatment, suggesting that mutated strains can escape from neutralizing antibodies, leading to higher rates of transmission [16,24,25,26]. The Omicron variant in particular has shown an increase in breakthrough infections in patients who are double vaccinated, illustrating the potential of this strain to evade neutralization [27].

With the potential for viral mutants to evade neutralization, researchers continue to focus on an ever-increasing range of methods to inhibit the SARS-CoV-2 virus in hopes of preventing further transmission. These include focusing on developing nanobodies or using monoclonal antibodies (mAbs) from COVID-19 patients [6,28,29,30], disrupting interactions with the furin cleavage site to prevent SPG priming and host cell entry [31], and targeting atypical portions of the virus such as the heptad repeat 1 (HR1) or the distal polybasic cleavage sites [32,33], along with an array of in silico studies to elucidate potential avenues of intervention [34,35,36,37,38,39,40,41]. Drug developers have also been investigating the effectiveness of existing antivirals and nucleoside inhibitors such as Triazivirin and Remdesivir, along with the recently produced Molnupiravir, which are inserted into nascent viral RNA and disrupt replication [42,43,44]. These therapeutic approaches show promise in reducing recovery time and risk of hospitalization in infected patients yet do not prevent the initial infection that can lead to further transmission before symptoms arise.

One avenue to further reduce transmission and host infection is through employing intelligent or “smart” textiles. These materials are wearable devices which actively monitor physiological conditions via biosensors embedded within the fabric of the garment [45]. Intelligent textiles have been adapted for the COVID-19 pandemic through advancements in nanotechnology and have been used to detect COVID-19 symptoms in patients through contactless processes [46,47]. These novel textiles are also being tailored to detect, trap, and neutralize potential pathogens or otherwise alert the wearer to their environmental presence through responses such as color change or flashing lights [48]. Further optimization of these fabrics, such as in face masks, through the use of integrated biosensors which target a specific virus or microorganism could influence how we approach the current and future pandemics.

Synthetic peptide design directed towards unsolved problems associated with viral transmission reduction provides a framework to consider inhibitory, capture, and detection solutions. For example, a better understanding of the binding requirements of peptides to SPG could inform intelligent textile design for the purpose of the capture, detection, and neutralization of viruses such as SARS-COVID-2 for improved personal protective clothing. Moreover, the use of bio-organic approaches to create surface detection of viruses through nanomaterial-immobilized peptides is of interest and has shown promise with dengue virus. However, approaches to mimicking the molecular features of host cell hot spot virus-binding motifs with synthetic peptides have, up until the COVID-19 pandemic, received scarce attention. Thus, the impetus for this study is to examine the conformational features of the helical ridge of the amino-terminal domain of ACE2 containing the characterized hot spot residues for SPG binding and to evaluate the relative role of the electrostatic charges associated with peptides designed to adopt an alpha helix. Previous work on peptide design in this regard has addressed the potential inhibition of SARS-CoV-2 and host cell receptor ACE2 fusion through tailored peptides and ACE2 derivatives that focus on optimizing hot spot interactions between the enzyme and the viruses’ spike glycoprotein RBD [49,50,51,52,53,54]. Additionally, other studies have investigated forcing conformational changes or steric hindrance in the virus SPG, which prevent the binding of RBD residues known to interact with the ACE2 N-terminus [28,55]. Much of this research has focused on relatively large analog design, i.e., twenty or more amino acids. However, few experimental approaches have reported minimal peptide sequences of less than fifteen amino acids that effectively bind to the SPG of SARS-CoV-2. Moreover, rationally designed peptides targeted to bind to the virus RBD can improve efforts to utilize virus fusion mechanisms and general protein–protein interactions (PPI) for new viral inhibitors, drugs, and detection approaches moving forward. Thus, we have examined synthetic peptides designed to evaluate putative binding to SARS-CoV-2 and its usefulness for virus capture, detection, and inhibition.

The secondary structural features of the amino-terminal ACE2-derived peptides were evaluated through circular dichroism (CD) measurements. Binding affinities to the SARS-CoV-2 spike protein RBD were assessed via surface plasmon resonance (SPR). We provide structure/function correlations from initially designed peptides that are based on co-crystallized ACE2/SARS-CoV-2 studies. In addition, we discuss a conformation- and charge-based synthesis approach to tailor peptides for potential virus capture by examining trends from SPG affinity binding and molecular docking simulations. The structure/function analysis is done with the aim of facilitating both virus inhibition and intelligent textile design to actively advance personal protective equipment [48,56].

## 2. Results

**Figure 1:**
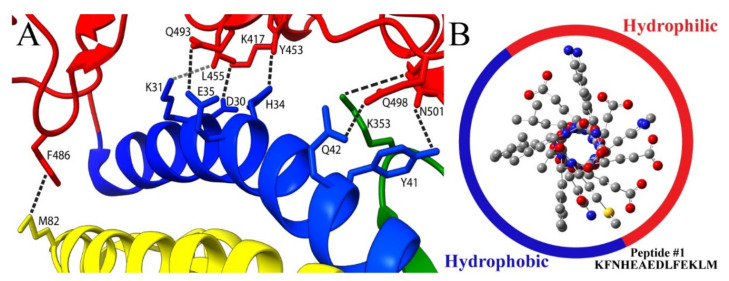
(**A**) SARS-CoV-2 spike RBD and human ACE2 complex based on the crystal structure. The blue, yellow, and green residues correspond to the ACE2 α1 helix, α2 helix, and β3 linker, respectively. The red residues are from the viral spike protein. Residues involved in the binding are noted, and interactions are illustrated via dashed line. The structure is visualized using ChimeraX software. (**B**) View of helical peptide 1 looking down the central axis as constructed in GaussView molecular modeling software. Hydrophobic residues lie on the left side of the helix, while the majority of hydrophilic residues are on the right. The hydrogen atoms have been removed to improve visibility of the helical structure and residue positioning.

In these studies of the interface between the SARS-CoV-2 spike RBD and human ACE2, residues that promote binding have been identified, similar to studies originally done on ACE2 binding with SARS-CoV [10]. Hydrophilic amino acids that reside on the α1 helix of hACE2 mediate this binding via polar interactions such as hydrogen bonding and salt bridges. Figure 1A illustrates this PPI between the SARS-CoV-2 spike protein RBD and human ACE2 based on the crystal structure (PDBid: 6M0J) of the complex. Residues that strongly contribute to the binding via hydrogen bonding and van der Waals forces are marked on the figure with the dashed line connecting them. The residues on the α1 helix, α2 helix, and β3 linker of ACE2 are colored blue, yellow, and green, respectively, while the SARS-CoV-2 RBD residues are shown in red. Additional residues associated with binding have also been identified for their interaction with the SPG [57,58,59,60].

### 2.1. Development of the Synthetic Peptides

The peptides of this study were designed to adopt a helical conformation with a classic amphiphilic alignment and balance of hydrophilic and hydrophobic residues based on the Chou–Fasman method [61]. Thus, the design paradigm is to mimic and reinforce the α1 helix structure of the amino-terminal portion of ACE2 that binds to SPG, as seen in previous structural studies [62]. The sequences are listed in Table 1, with the bold red residues noting portions of the sequences which correspond to the α1 helix of human ACE2. Figure 1B illustrates this design motif when Peptide 1 adopts a helical conformation, placing the hydrophobic residues of Ala, Phe, Leu, and Met reside mostly on the left side of the helix, while the hydrophilic residues Lys, Asn, His, Glu, and Asp are mainly on the right.

### 2.2. Circular Dichroism Measurements and Secondary Peptide Structure

**Figure 2:**
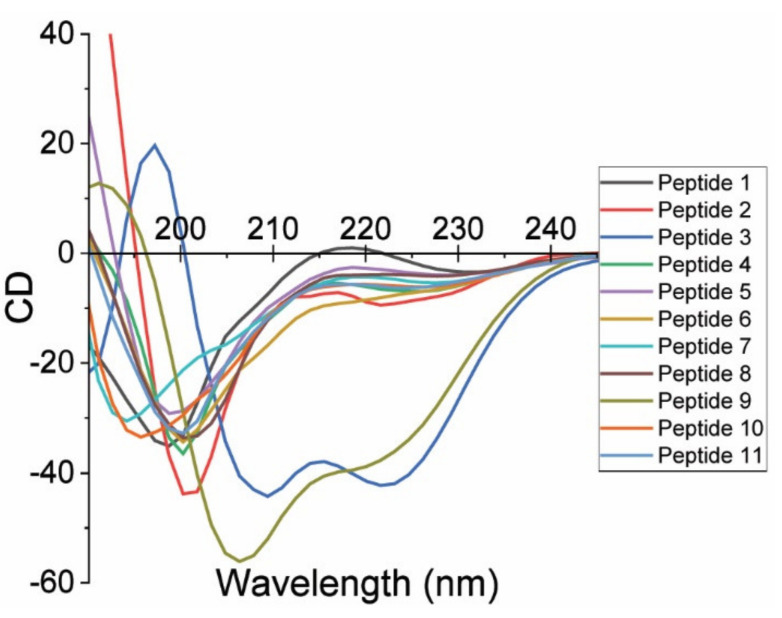
Circular dichroism measurements for eleven synthetic peptides in PBS-T (pH 7.4).

The secondary structure of the peptides was measured to discern which analogs adopt a helical structure under aqueous conditions. Figure 2 presents the circular dichroism (CD) measurements for the eleven synthetic peptides in PBT-S (pH 7.4). The spectra are consistent, with most of the analogs adopting a random coil structure. This result is based on the most significant spectral feature being the trough at 190–200 nm, a characteristic of a π → π* transition which is expected for peptides with ten or more amino acids in the polymer backbone chain [63]. This is illustrated in Peptides 1, 2, 4, 5, 7, 8, 10, and 11. The similarities between these CD results are understandable, as most of the peptide analogs share sequences with some functional variation at end-group modifications. Peptides 3 and 9 (blue and yellow lines) are the only samples which show characteristics of an alpha helix based on the negative peak at 225 nm, a function of the n → π* transition, and the positive and negative peaks around 190 and 210 nm, resulting from amide bond exciton splitting of the π → π* transition [64]. Peptide 3 is a head-to-tail cyclic analog that would prevent unfolding and further stabilize a helical structure. While Peptide 6 (orange line) is a cyclic analog, formed from a Cys–Cys disulfide bridge, it shows a CD spectrum characteristic of a cyclized peptide [65], but does not appear to adopt a helical structure. Thus, given the analogy between the two cyclic analogs, the stabilization of the helical structure observed in analog 3 appears to result from the retention of a contiguous amide backbone structure, and analog 6 retains the amino- and COOH-terminal functionality with insufficient stabilization of the backbone chain to stabilize the helix under aqueous conditions.

### 2.3. Binding Kinetics

**Figure 3:**
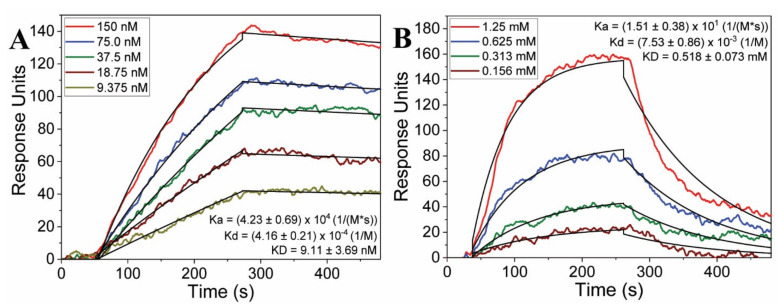
(**A**) Binding kinetics between spike glycoprotein and ACE2, and (**B**) kinetics measuring the binding between the spike protein and Peptide 3. The fit lines are shown in black for each curve, and the binding kinetics values (Ka, Kd, and KD) for each sample series are provided.

Binding studies initially assessed human ACE2 protein as the analyte and the SARS-CoV-2 spike protein RBD as the ligand to provide a baseline for comparison. The results from the PPI binding study are shown in Figure 3A for ACE2 samples ranging in concentration from 9 nM to 150 nM. A detailed explanation of the experimental method is provided in the Materials and Methods section. The figure shows clear binding and dissociation between the ligand (SARS-CoV-2 spike protein RBD) and analyte (human ACE2) and is fit well by the 1:1 binding kinetics model, represented by the black line on each curve.

From the results of the fit, the association (Ka) and dissociation (Kd) values are determined to be (4.23 ± 0.69) × 10^4^ (1/(M*s)) and (4.16 ± 0.21) × 10^−4^ (1/s), respectively, and the overall KD is 9.11 ± 3.69 nM. These values are similar to previously reported data for SPG and ACE2 binding which showed a maximum binding strength of approximately 20 nM [66].

The binding capabilities of the peptide analogs were measured against the SARS-CoV-2 SPG RBD. Peptides 1–7 and 11 were designed with the aim of reinforcing the amino-terminal α1 helix of ACE2 to facilitate binding. Peptide 7 also contains an EEL(F/W)E sequence, as do Peptides 8 and 10. Notably, the EEL(F/W)E motif has been proposed to bind to a polybasic binding region of the SPG [33]. Peptide 9 was previously characterized by its helical conformation with minimal sequence requirements, and is included for relevance to that design feature [63]. The results from these SARS-CoV-2 binding experiments are provided in Table 2. Ten analogs showed measurable binding within the concentration range. Notably, Peptide 11 also bound to the spike protein RBD at higher concentrations, but solubility issues prevented suitable measurements.

Head-to-tail cyclization of ACE2 (31–40)Glu-41,Lys-42,Leu-43)Met(82) (Peptide 3) results in an order of magnitude increase in binding affinity, with a KD around 500 µM. The peptides were measured in multiple iterations to confirm the results, and Figure 3B presents one experimental trial for the Peptide 3 binding series to the SPG RBD. Peptide 3 solutions ranged from 0.156 to 1.25 mM, and the Ka and Kd were determined to be (1.51 ± 0.38) × 10^1^ (1/(M*s)) and (7.53 ± 0.86) × 10^−3^ (1/s), respectively, with a binding affinity (KD) of 518 ± 73 µM. The corresponding fit based on a 1:1 binding ratio is given in the figure (black lines). The full details of the measurements are provided in Materials and Methods.

Interestingly, Peptide 3 was also one of two analogs which showed a helical secondary structure in the CD results. Research investigating the structural conformation of peptide binding profiles to the SPG of SARS-CoV-2 has shown that a helical structure produces stronger binding to the spike protein RBD compared to an unstructured/unfolded peptide [40].

There is a trend with some of the peptide analogs that is based on the N-terminal helical ridge of ACE2. In this regard, Peptides 1, 2, 4–8, and 10 demonstrate a random coil secondary structure in the CD measurements (Figure 2), and these analogs demonstrated binding affinities between 1.4 and 43 mM. The differences in binding strengths are attributed to changes in end-group and sequence residues. This relationship is discussed further below, with considerations of the effect of the charge of both the peptides and SPG variants. Prior studies investigating the binding of synthetic peptides based on ACE2 hot spots produced a Kd similar to the binding constant results of this study [51,58,67]. While previous studies used a variety of ACE2-derived peptides, none were reported to be characterized with cyclically stabilized and minimal sequence structures, i.e., less than twenty amino acids.

Peptide 3 is cyclized and stabilized as a helical structure, yielding a binding concentration at least 3-fold higher than the analogous linear peptides (1, 2, 4, and 5). This is consistent with previous reports on stabilized peptide helices representing ACE2 [51,52,53]. However, our study is the first report to date utilizing a decatetrapeptide (14 amino acids) with helical stability and micromolar affinity.

Importantly, cyclized peptides have been efficacious in directing drug delivery and therapeutics due to their enhanced chemical and proteolytic stability and have shown more selective binding modes when compared to analogous sequences with unordered structure [68,69,70]. Historically, cyclic peptides have produced enhanced activity when examined in the context of naturally occurring linear peptide receptor ligands [71]. Moreover, the first reported synthetic neuropeptide was created through a disulfide cyclization. Much of the early work on neuropeptide structure/function relations led to therapeutic potential. These peptides have been shown to undergo changes upon either disulfide or amide bond cyclization, which produce an enhanced ability to orient side chains and the amino acid backbone toward target receptors, increasing binding affinity and selectivity [72,73]. It is notable as well that a recent report of a decatetrapeptide (14 amino acid) cyclic analog (AMY-101 TFA) directed to complement associated pathology exhibited efficacious treatment in COVID-19 pneumonia patients with severe inflammation [74]. This is consistent with a minimal peptide sequence that would confer sufficient binding affinity to exert therapeutic efficacy by way of an enzyme or receptor protein.

### 2.4. Modeling Considerations

**Figure 4:**
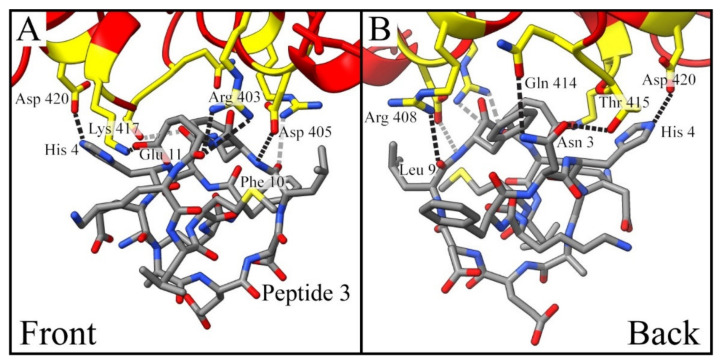
The molecular docking modeling of the SARS-CoV-2 spike protein RBD with Peptide 3. The spike protein is colored red with the nine residues that could provide avenues for binding to the peptide highlighted in yellow. Heteroatoms are colored accordingly on the highlighted residues and the peptide sequence. The hydrogen atoms have been removed for clarity. View of the docking shown from the front (**A**) and back (**B**) as illustrated in ChimeraX software. The black dashed lines represent the interactions in the foreground of the image, while the gray dashed lines are in the background.

**Figure 5:**
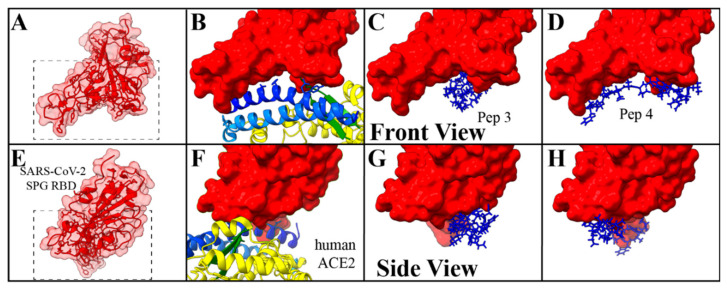
Molecular docking studies with SARS-CoV-2 SPG RBD. Zoomed out front (**A**) and side (**E**) perspective of the RBD, with the extent of the electrostatic potential surface shown in transparent red. A dashed box is provided which highlights the window used in the other images (**B**–**D**,**F**–**H**). The results of the docking simulation between the SARS-CoV-2 spike glycoprotein RBD and human ACE2 (**B**,**F**), Peptide 3 (**C**,**G**), and Peptide 4 (**D**,**H**) are provided for each perspective, with the RBD surface presented in solid red.

Molecular docking simulations were performed to better understand the binding mechanisms for the peptides with the virus SPG. A close-up model for the results of the calculation between SARS-CoV-2 RBD and Peptide 3 is shown in Figure 4, providing front (Figure 4A) and back (Figure 4B) perspectives. Seven RBD residues which interact with Peptide 3 are highlighted in yellow, while the remainder of the molecular structure is red. The SPG residues and corresponding interacting peptide functionalities include: Arg 403 → Glu 11 (×2), Asp 405 → Phe 10, Arg 408 → Leu 9, Gln 414 → Asn 3, Thr 415 → Asn 3, Lys 417 → Glu 11, and Asp 420 → His 4. Notably, the conformationally constrained nature of the cyclic peptide structure permits the interaction of the SPG with both the hydrophilic and hydrophobic faces of Peptide 3 consistent with the occupation of the constrained 14-mer in a cleft of the SPG protein structure (Figure 5C,G) adjacent to the RBD. This result suggests that there are numerous putative binding sites on the RBD that are accessible to the cyclic 14-mer. It is necessary to note that while the CD measurements for Peptide 3 suggest that it has an alpha helical secondary structure, the minimized geometry performed through the docking simulations does not. While the cyclic peptide winds around itself twice in a screw-like fashion, the phi and psi angles are not representative of the definition of an alpha helix. It is very possible the process of cyclization distorted the original helical structure, yet these distortions appear as an alpha helix in the CD measurements. Further characterization of the peptide would be required to know the precise structure.

The three-dimensional (3D) space occupied by the peptides was compared to that of human ACE2. Figure 5 depicts a larger view of docking models for the binding of the spike protein RBD to human ACE2 and to the two peptides which have the strongest binding in this study, Peptide 3 (KD = 518 µm) and Peptide 4 (KD = 1370 µm). These images are presented in a front view (Figure 5A–D) and a side view (Figure 5E–H) where the structure is rotated 90° to provide a better idea of the 3D interactions. Each set of views is precluded by a zoomed-out perspective of the SPG RBD (Figure 5A,E), which highlights within the dashed box the distal portion of the virus which interacts with human ACE2 and the peptides. The extent of the electrostatic surface for the SARS-CoV-2 spike glycoprotein RBD is shown in transparent red for the zoomed-out view and solid red for the docking simulation results. The important structural features and known binding residues of human ACE2 (Figure 5B,F) are shown in the same color scheme as in Figure 1. As can be seen from these structures, Peptides 3 (Figure 5C,G) and 4 (Figure 5D,H) occupy similar binding motifs as ACE2 and interact with the same residues of RBD sequence. Table 3 provides a list of the Peptide 3 and 4 residues which interact with the spike protein RBD based on the docking simulations. Residues highlighted in red are those which coincide with the fifteen known ACE2–SPG RBD binding locations [57,59]. Additionally, the distances of these interactions were measured and are provided in the table next to each binding entry.

Peptide 3 has the strongest binding with the spike protein RBD. The molecular modeling demonstrates the interaction of one of the residues as being identical to the ACE2–SARS-CoV-2 binding (Lys 417). Other putative residue interactions are noted. It is evident that the cyclic peptide constraints minimize the molecular model to a conformation that fits in a region of SPG protein that overlaps the RBD yet binds some residues that have hitherto been unidentified in the binding of ACE2 to SPG. On the other hand, Peptide 4, which is also based on the ACE2 α1 helix with an unordered secondary structure, binds to ACE2 via interactions with the RBD that are consistent with the native protein structures depicted in Figure 1A. Based on the docking simulations, the interactions between Peptide 4 and the spike protein RBD principally occur at the terminal portions of the peptide sequence.

### 2.5. Residue Substitution and Electrostatic Effects

**Figure 6:**
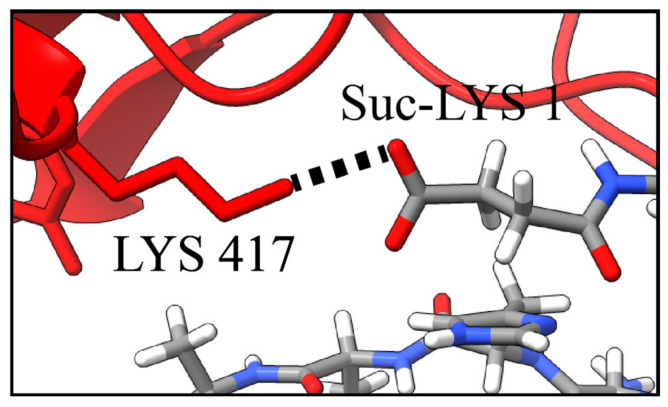
Interaction between the viral spike protein RBD residue (Lys 417) with the succinyl group of modified Lys 1 on Peptide 4. Results are based on molecular docking calculations.

A goal of the structure/function assessment is to investigate the relative effect of amino- and COOH-terminal modifications on binding to guide future synthetic peptide studies. A trend based on the effect of charge is apparent from the experimental results (Table 2). Specifically, for the N-terminus of the peptides, the end-group substitutions resulting in the strongest to weakest binding affinity proceed as succinylation > H > acetylation. Likewise, the C-terminal substitutions proceed as OH > NH_2_. Both trends suggest that greater end-group electronegativity produces stronger binding. Notably, succinylation of a lysine end group reverses the charge of lysine from its (+1) to a (−1) under physiological conditions, while acetylation removes the charge, resulting in a neutral species [75]. An example of this binding interaction is noted in Table 3 and illustrated in Figure 6, showing the succinylated lysine residue of Peptide 4 in close proximity (2.8 Å) with Lys 417 of the spike glycoprotein RBD. Lys 417 has been identified as a residue which significantly contributes to the binding between the SARS-CoV-2 virus and human ACE2 [59]. Thus, succinylation of the amino-terminus of Peptide 4 is important to consider.

Though amino acid sequence similarities are inherent to the conformation relations of this work, the binding affinities show significant variations attributable to end-group modifications. For example, Peptide 1, which comprises the basic sequence native to the amino-terminal ACE2 host cell receptor, is a truncated analog that includes the hot spot binding residues of Lys 31, His 34, Glu 37, and Asp 38 [59,60]. Peptide 2 shares the same basic residue sequence but with a COOH-terminal amidation, which results in a circa 10-fold decrease in binding affinity. However, Peptide 4 contains both the COOH-terminal amide modification and N-terminal succinylation which results in a 2-fold increase in binding affinity compared to Peptide 1 and a nearly 20-fold increase compared to that of Peptide 2.

Consistent with the theme of electrostatic effects on binding, the isoelectric point (pI) for each analog was calculated, and these values are noted in Table 1. At the isoelectric point of a peptide, the net charge is zero, while the net charge is negative at a pH above the pI. The peptides in this study have a pI between 3.9 and 5.4, suggesting that they have a net negative charge notwithstanding residue modification. Succinylation and amino acid substitutions, such as glutamic acid, further increase the negative charge of the peptide structure. Examination of the effect of end-group modification and substitution reveals an overall increase in the binding affinity of the peptides to the spike protein RBD as the overall charge of the peptide becomes more negative. These results highlight the importance of charge in facilitating binding between synthetic peptide analogs and the viral SPG and informs future approaches designed to enhance this interaction.

### 2.6. Consideration of the Influence of Charge at the RBD of SARS-CoV-2 Variants

Based on the apparent effect of peptide charge on binding affinity, we surveyed possible trends in the SPG electrostatic differences of virus variants. Research investigating ACE2 binding to the SARS-CoV and SARS-CoV-2 RBD shows that the sequence binding residues from the former to the latter result in stronger binding due to increased electrostatic potential and van der Waals interactions. These are in part attributed to the increased virulence of SARS-CoV-2 [10]. While many of the sequence changes from SARS-CoV to SARS-CoV-2 replace neutrally charged residues with other neutral residues, two specific changes, V404 → K417 and D480 → S456, effectively cancel out the introduction of a negatively charged residue substitution and shift the overall charge of the RBD to a net positive value [10].

Similar trends are seen with mutations from the SARS-CoV-2 WT to viral variants [21,24,76,77]. These mutations are outlined in Table 4 and illustrate that each contains residue substitutions which shift the overall charge of the spike glycoprotein to a less negatively or more positively charged structure under physiological pH. In fact, recent research has shown that certain mutations directly affect the binding affinity between SARS-CoV-2 variants and human ACE2. The mutations which increase binding, E484K and L452R and combinations of mutations including these, provide a net positive shift to the SARS-CoV-2 electrostatic potential, while a notable mutation which decreases binding affinity, K417N, shifts the overall charge negatively [78]. A recent study by Fantini et al. provided an index of the transmissibility metric for viral variants which examined changes in the RBD electrostatic potential via residue mutations. The results correlate increased virulence with mutations garnering a net positive change to the RBD electrostatic surface [79]. While likely not the main contributor to the higher binding affinities and increased transmission rates of the variants, as residue mutations may be accompanied with conformational changes which affect binding interactions [80], the charge shift should not be dismissed as a significant factor in the protein–protein interactions.

The Delta variant accounted for nearly all new COVID-19 cases in the United States in the Fall of 2021 [81]. However, with the emergence of the highly mutated Omicron variant, this strain of the SARS-CoV-2 virus has become the predominant mutant worldwide, owing its increased virulence to the more than 30 residue mutations on its spike protein [82]. Of note are the Q493R and Q498R mutations, which have been shown to increase binding affinity with human ACE2. These mutations correspond to positive charge changes and allow for the formation of new salt bridges and hydrogen bonds, while other mutations improve antibody evasion [20,83]. The one commonality with the variants of concern is that the overall mutations have the effect of increasing the net charge, yielding a more net positive structure (Table 4). Therefore, coupling the positive electrostatic potential of the spike protein RBD with an increasing negative charge in peptide and analogous design systems could, in theory, facilitate a stronger binding affinity for viral capture, detection, or inhibition design motifs moving forward.

## 3. Materials and Methods

### 3.1. Peptide Design and Synthesis

Elected peptide sequences of this study were envisioned and optimized as truncated peptide analogs of human ACE2. ACE2 amino-terminal peptide analogs were designed through an examination of the binding epitopes of co-crystalized structures of ACE2 and SARS-CoV-2 spike glycoprotein [84], and the sequences advanced from their Cambridge Crystal structure and PDB structure through crystal coordinate acquisition and extraction to a graphical disposition of the peptides in helical wheel design motifs ranging from 14 to 21 amino acids. For example, the basic 14-mer is ACE2(31–40)EKLM(82), so the first ten residues from the amino terminus are identical to ACE2 31–40 and residues 11, 12, and 13 are Glu, Lys, Leu, and the COOH terminal residue methionine is modeled from Met 82 of ACE2. Thus, the fourteen amino acid analog Peptide 1 represents the primary hot spot residues of ACE2 that have been correlated with binding to the receptor-binding domain of the SPG. Peptides were further optimized while remaining consistent with the amino-terminal helical ridge of ACE2(31–40) and inclusive of the hot spot region of the ACE2 receptor-binding domain. Chou–Fasman rules were applied to optimize alpha helical design motifs with an amphiphilic residence of helix-promoting residues to balance charge and promote stabilization of conformation [61]. Cyclization, amino-terminal, and COOH-terminal modification protocols were applied to examine modulation of charge and promote a stabilized conformation. Thus, eleven peptides were subsequently synthesized at Biomatik (Wilmington, DE, USA) and chromatographically confirmed to be greater than 95% pure by HPLC. The specific sequence for each peptide and the resulting mass spectroscopic parent ion are provided in Table 1. The highlighted residues correspond to a portion of the α1 helix sequence of human ACE2 from K31 to F40. Note that Peptides 3 and 6 are cyclic analogs of ACE. The other nine linear analogs contain ACE2 hot spot amino acids in register, with amphiphilic helices in alignment with the binding epitopes. Table 5 provides the results of the mass spectrometry characterization and HPLC-determined purity for each peptide, showing that the samples are greater than 95% pure.

### 3.2. Recombinant Proteins

The recombinant SARS-CoV-2 spike glycoprotein receptor-binding domain and recombinant human ACE2 were purchased from Sino Biological (Wayne, PA, USA). The spike protein was expressed via baculovirus–insect cells and polyhistidine (His tag) added to the C-terminus to allow for attachment to the SPR sensor surface. The ACE2 sample was expressed through HEK293 cells and has an hFc tag on the C-terminus. The SARS-CoV-2 spike glycoprotein and human ACE2 samples are confirmed to be greater than 95% pure, as determined by SDS-PAGE (Sino Biological, Wayne, PA, USA).

The recombinant streptavidin, purchased through Amid Biosciences (Santa Clara, CA, USA), was expressed via *E. coli* and contains a His tag on the C-terminus for attachment to the surface as a blocking agent in the OpenSPR instrument reference channel.

### 3.3. Peptide Circular Dichroism Measurements

Eleven samples in solution were produced from the peptides. An amount of 3 mg of each peptide was placed in 5 mL of pH 7.4 phosphate-buffered saline with 0.05% Tween20 (PBS-T) buffer, resulting in 0.6 mg/mL solutions for each. The secondary structures of the peptides were evaluated by circular dichroism spectroscopy (Olis RSM 1000) (Olis, Athens, GA, USA) using a cylindrical cuvette with 1 mm path length at 25 °C. CD spectra were obtained from 185 to 260 nm at a scanning speed of 75 nm/min with 1.54 nm increments and 0.3 s integration time. The resulting CD spectra were analyzed in OriginPro software (OriginLab Corporation, Northampton, MA, USA) and smoothed via Savitzky–Golay method, using 6 adjacent points for averaging.

### 3.4. Surface Plasmon Resonance (SPR) Measurements

The binding measurements were performed on a Nicoya (Kitchener–Waterloo, ON, Canada) OpenSPR 2-channel instrument. The nitrilotriacetic acid (NTA) sensor was pretreated with 10 mM HCl, 350 mM EDTA, and 40 mM NiCl_2_ to prepare the surface for ligand attachment. The running buffer for ligand attachments and all measurements was PBS-T. The SARS-CoV-2 spike glycoprotein receptor-binding domain was the ligand for these experiments and attached noncovalently to the NTA sensor surface via the C-terminus His tag in the OpenSPR second channel. The spike protein sample was injected at a flow rate of 20 µL/min and a concentration of 40 µg/mL. After S protein attachment, streptavidin was injected into both channels and attached to the surface via His tag to act as a negative control protein and reduce non-specific binding. The streptavidin was injected at a flow rate of 20 µL/min and a concentration of 50 µg/mL.

Binding experiments to the spike protein were performed with samples of ACE2 and the synthetic peptides. The concentration for the ACE2 samples ranged from 9 to 150 nM, whereas the peptides had much larger concentrations of 60 µM to 5.0 mM. The binding for each sample was measured from lowest to highest concentration at a flow rate of 20 µL/min, and each trial was performed in triplicate. If necessary, a solution of 1.0 M MgCl_2_ was injected between measurements to remove any bound samples and regenerate the surface.

The binding kinetics for each series of measurements were analyzed using TraceDrawer software with a 1:1 binding ratio. The variables for analysis are as follows: BI was set to “Constant” because bulk shift is subtracted by the reference channel, ka and kd were set to “Global”, and Bmax was set to “Local” because the ligand was not fully regenerated between analyte injections. Multiple trials were performed for each concentration series, and the average and standard deviation for each sample were calculated from the results.

### 3.5. Molecular Docking Simulations

Docking studies were performed in Molecular Operating Environment (MOE) software version MOE 2020.0901 (Chemical Computing Group ULC, Montreal, QC, Canada). The SARS-CoV-2 spike protein RBD domain was prepared using the crystal structure of the RBD-hACE2 receptor complex (PDB 6M0J). After removing hACE2 from the complex, the RBD protein was cleaned and minimized. Cyclic peptides were built in MOE with the protein builder tool and minimized. Minimizations were accomplished with AMBER10:EHT forcefield settings, with an RMS gradient of 0.1 kcal/mol/Å^2^. The RBD protein was chosen as the binding site and receptor site; for a given docking run, the respective cyclic peptide was chosen as the ligand. For each cyclic peptide, two docking experiments were performed: once using the “Rigid Receptor” refinement setting, and once using the “Induced Fit” refinement setting. The placement setting was set to “Triangle Matcher” for every run. Thirty poses were generated from each docking study with London ΔG scoring applied and further refined with GBVI/WSA ΔG scoring to obtain five final poses. ChimeraX was then used to visualize the generated poses of the peptides in complex with the RBD domain.

## 4. Conclusions

SARS-CoV-2, the coronavirus that is the causative agent of the COVID-19 pandemic, is continuing to spread around the globe. While there is hope of the pandemic coming to an end by way of a multitude of vaccines and the potential for effective oral medications, the highly transmissible nature of the virus suggests that innovative routes of inhibiting the spread are still needed. We set out to identify molecular motifs with synthetic peptides containing amino acid substitutions that specifically target the SARS-CoV-2 SPG. The goal of this approach is to provide a framework for new molecular constructs useful in virus capture, detection, and inhibition with the aim of employing minimal sequence requirements for binding. Synthetic peptides based on the structure of human ACE2 binding hot spots present a potential avenue for this approach. The results reported take into consideration additional factors that expand on previous reports and illustrate small-peptide binding affinities in the µM range. Stabilizing the helical structure through cyclization improves the binding constant by an order of magnitude compared to the unfolded sequences. Additionally, using charged species, whether at the end groups of the peptides or by substituting differently charged residues, appears to further improve receptor-binding interactions. While these binding affinities may not be strong enough to competitively bind to SARS-CoV-2 in the presence of ACE2, using cyclic peptides as a lead and optimizing future structures through judicial modification and substitutions could increase the interaction enough for the proposed analogs to further viable antiviral candidate development. Furthermore, attaching small peptides or peptidomimetics to textiles such as clothing and face masks presents an opportunity to develop a bio-organic approach to intelligent textiles that actively capture and detect the virus. This approach is considered viable for inclusion in the potential armamentarium to inhibit viral transmission.

## Figures and Tables

**Table 1:** Synthetic peptide sequences and calculated isoelectric points for each sample.

Sample	Sequence—Head to Tail (N to C)	Isoelectric Point (pI)
1	KFNHEAEDLFEKLM-OH	4.6
2	KFNHEAEDLFEKLM-NH_2_	5.4
3	KFNHEAEDLFEKLM (head-to-tail cycle)	4.6
4	Suc-KFNHEAEDLFEKLM-NH_2_	5.4
5	Ac-KFNHEAEDLFEKLM-OH	4.2
6	CKFNHEAEDLFEKLMC-NH_2_ (S=S bridge)	4.5
7	Suc-KFNHEAEDLFEKLFEELFEDM-NH_2_	4.2
8	GLFKEALEELWEA-NH_2_	4.3
9	Suc-LLEKLLEWLE-NH_2_	4.6
10	KFNDEAEDLFKLFEELFEDM-NH_2_	3.9
11	MIEEQAKFLDKFNHEAEDLFK-NH_2_	4.8

Highlighted red residues denote residues which correspond to N-terminal “hot spots” of human ACE2.

**Table 2:** Binding kinetics of synthesized peptides to SARS-CoV-2 spike protein RBD.

Peptide Number	Bulk Concentration (mM)	Binding (Yes/No)	Ka (M^−1^ s^−1^)	Kd (M^−1^)	KD (mM)
1	5.0	Yes	4.62 ± 0.45	(1.33 ± 0.27) × 10^−2^	2.84 ± 0.30
2	5.0	Yes	1.15 ± 0.08	(2.58 ± 0.80) × 10^−2^	23.1 ± 0.84
3	1.25	Yes	15.1 ± 3.8	(7.53 ± 0.87) × 10^−3^	0.518 ± 0.073
4	5.0	Yes	13.9 ± 3.6	(1.87 ± 0.39) × 10^−2^	1.37 ± 0.07
5	5.0	Yes	0.64 ± 0.09	(3.38 ± 0.06) × 10^−2^	42.9 ± 2.4
6	2.5	Yes	6.84 ± 2.46	(1.12 ± 0.08) × 10^−2^	1.83 ± 0.54
7	1.25	Yes	4.29 ± 0.52	(2.09 ± 0.39) × 10^−2^	5.05 ± 0.15
8	2.0	Yes	5.39 ± 1.31	(1.19 ± 0.01) × 10^−2^	2.53 ± 0.58
9	2.5	Yes	5.83 ± 2.92	(1.39 ± 0.16) × 10^−2^	3.37 ± 1.9
10	1.0	Yes	6.81 ± 2.41	(1.75 ± 0.46) × 10^−2^	2.66 ± 0.28
11	1.0	No	−	−	−

**Table 3:** Binding sites between Peptides 3 and 4 and the spike protein RBD.

Peptide 3		Peptide 4	
Residue Interaction	Distance (Å)	Residue Interaction	Distance (Å)
Arg 403 → Glu 11 *	2.9	Arg 403 → Glu 7	3.5
Arg 403 → Glu 11 *	3.5	Asp 405→ Met 14-NH_2_	3.1
Asp 405 → Phe 10 *	2.6	Lys 417 → Suc-Lys 1	2.8
Arg 408 → Leu 9 *	3.2	Gly 496 * → Asp 8	3.2
Gln 414 → Asn 3	2.6	Asn 501 → Leu 9 *	3.2
Thr 415 → Asn 3	2.9	Val 503 → Leu 13 *	3.1
Lys 417 → Glu 11	3.2	Tyr 505 → Glu 7	2.9
Asp 420 → His 4	3.7	Tyr 505 → Glu 7	2.9

Red residues denote amino acids with which human ACE2 interacts as well. * Denotes residue interaction occurs on the amino acid backbone instead of side chain.

**Table 4:** SARS-CoV-2 spike protein sequence mutations by variant of concern.

Variant (Pango Lineage)	Spike Mutation	Residue Charge Change	Net Charge Change
Alpha (B.1.1.7) Overall charge change: Positive	D178H	(−) to neutral	(+)
	E484K	(−) to (+)	(++)
	N501Y	neutral to neutral	no change
	A570D	neutral to (−)	(−)
	P681H	(+) to neutral	(−)
	T716I	neutral to neutral	no change
	S982A	neutral to neutral	no change
	D1118H	(−) to neutral	(+)
Beta (B.1.351) Overall charge change: Positive	L18F	(+) to neutral	(−)
	D80A	(−) to neutral	(+)
	D215G	(−) to neutral	(+)
	R246I	(+) to neutral	(−)
	K417N	(+) to neutral	(−)
	E484K	(−) to (+)	(++)
	N501Y	neutral to neutral	no change
	D614G	(−) to neutral	(+)
	A701V	neutral to neutral	no change
Gamma (P.1) Overall charge change: Positive	L18F	neutral to neutral	no change
	T20N	neutral to neutral	no change
	P26S	neutral to neutral	no change
	D138Y	(−) to neutral	(+)
	R190S	(+) to neutral	(−)
	K417T	(+) to neutral	(−)
	E484K	(−) to (+)	(++)
	N501Y	neutral to neutral	no change
	D614G	(−) to neutral	(+)
	H655Y	neutral to neutral	no change
	T1027I	neutral to neutral	no change
Delta (B.1.617.2) Overall charge change: Positive	T19R	neutral to (+)	(+)
	V70F	neutral to neutral	no change
	T95I	neutral to neutral	no change
	G142D	neutral to (−)	(−)
	E156-	remove (−)	(+)
	F157-	remove neutral	no change
	R158G	(+) to neutral	(−)
	A222V	neutral to neutral	no change
	W258L	neutral to (+)	(+)
	K417N	(+) to neutral	(−)
	L452R	neutral to (+)	(+)
	T478K	neutral to (+)	(+)
	D614G	(−) to neutral	(+)
	P681R	neutral to (+)	(+)
	D950N	(−) to neutral	(+)
Omicron (B.1.1.529) Overall charge change: positive	A67V	neutral to neutral	no change
	T95I	neutral to neutral	no change
	G142D	neutral to (−)	(−)
	L212I	neutral to neutral	no change
	G339D	neutral to (−)	(−)
	S371L	neutral to neutral	no change
	S373P	neutral to neutral	no change
	S375F	neutral to neutral	no change
	K417N	(+) to neutral	(−)
	N440K	neutral to (+)	(+)
	G446S	neutral to neutral	no change
	S477N	neutral to neutral	no change
	T478K	neutral to (+)	(+)
	E484A	(−) to neutral	(+)
	Q493R	neutral to (+)	(+)
	G496S	neutral to neutral	no change
	Q498R	neutral to (+)	(+)
	N501Y	neutral to neutral	no change
	Y505H	neutral to neutral	no change
	T547K	neutral to (+)	(+)
	D614G	(−) to neutral	(+)
	H655Y	neutral to neutral	no change
	N679K	neutral to (+)	(+)
	P681H	neutral to neutral	no change
	N764K	neutral to (+)	(+)
	D796Y	(−) to neutral	(+)
	N856K	neutral to (+)	(+)
	Q954H	neutral to neutral	no change
	N969K	neutral to (+)	(+)
	L981F	neutral to neutral	no change

**Table 5:** Parent ion for each synthetic peptide, calculated molecular weight, and purity.

Peptide	Calculated MW (g/mol)	Parent Ion (*m*/*z*) *	Purity by HPLC (%)
1	1750.97	876.35 [2+]	97.17
2	1749.98	876.00 [2+]	97.51
3	1732.97	865.40 [2−]	95.59
4	1850.07	925.95 [2+]	97.19
5	1793.00	897.40 [2+]	97.07
6	1954.27	978.10 [2+]	96.49
7	2759.99	1380.90 [2+]	95.86
8	1533.76	1534.65 [1+]	96.62
9	1384.61	1385.60 [1+]	96.40
10	2508.75	1256.35 [2+]	96.62
11	2581.89	1291.85 [2+]	96.34

* Number provided in brackets for each entry is the associated charge of each ion.

## Data Availability

Data available from authors upon request.

## Sample Availability

Samples of the compounds are not available from the authors.
